# Rolling stones and turbulent eddies: why the bigger live longer and travel farther

**DOI:** 10.1038/srep21445

**Published:** 2016-02-17

**Authors:** Adrian Bejan

**Affiliations:** 1 Duke University, Durham, NC 27708-0300, USA

## Abstract

Here we report the discovery that even the simplest, oldest and most prevalent forms of evolutionary movement—rolling bodies and whirls of turbulence—exhibit the same body-size effect on life time and life travel as the evolutionary movement united by the body-size effect so far: animals, rivers, vehicles, jets and plumes. In short, the bigger should last longer and travel farther. For rolling bodies, the life span (t) and the life travel (L) should increase with the body mass (M) raised to the powers 1/6 and 1/3, respectively. The number of rolls during this movement is constant, independent of body size. For an eddy of turbulence, t should increase with the eddy mass (M) raised to the power 2/3, while L should increase with M^2/3^ times the bulk speed of the turbulent stream that carries the eddy. The number of rolls during the eddy life span is a constant independent of eddy size.

Recent progress on the physics basis of evolutionary organization in nature^1^,^2^,^3^,^4^,^5^,^6^,^7^,^8^,^9^,^10^ continues to bring together phenomena and explanations that previously were considered resolved and unrelated. To the wide diversity of animate and inanimate examples (animal locomotion, river basins, snowflakes, respiration, city sizes) that were unified as a phenomenon of free-morphing flow configuration toward greater access over time, we are now seeing examples that were considered understood, in well-established domains of science. One such example—the life span phenomenon—is the subject of the present report.

It is well known that bigger animals live longer. In biophysics, numerous observations have been correlated into power-law formulas indicating that the life span (t) increases with the body mass (M) approximately in proportion with M^γ^, where for mammals the γ varies in the range 0.2–0.22^11^,^12^,^13^,^14^,^15^,^16^,^17^,^18^. In a recent report^19^ these observations were generalized in two ways:

First, the body-size effect on life span was predicted theoretically (with γ = 1/4), as a consequence of the constructal law, which previously was shown to account for the body-size effect on all forms of animal locomotion^20^.

Second, the body-size effect that is documented and correlated in biophysics has very clear manifestations in two other realms: inanimate flow architectures (river basins, jets and plumes), and human-made vehicles. In all such cases, the bigger flows last longer and travel farther. The predicted life span and range of vehicles was shown to be the basis for technology evolution, for example, the evolution of commercial aviation, as an illustration of the evolution of human & machine movement on the globe^21^.

In the present report, we will see that the physics of the body-size effect on life span unites not only the animate, the inanimate and the technological examples recognized above, but also the simplest and oldest imaginable forms of evolutionary movement on earth: rolling stones and rolling eddies in turbulent flow.

## Results

**Figure 1:**
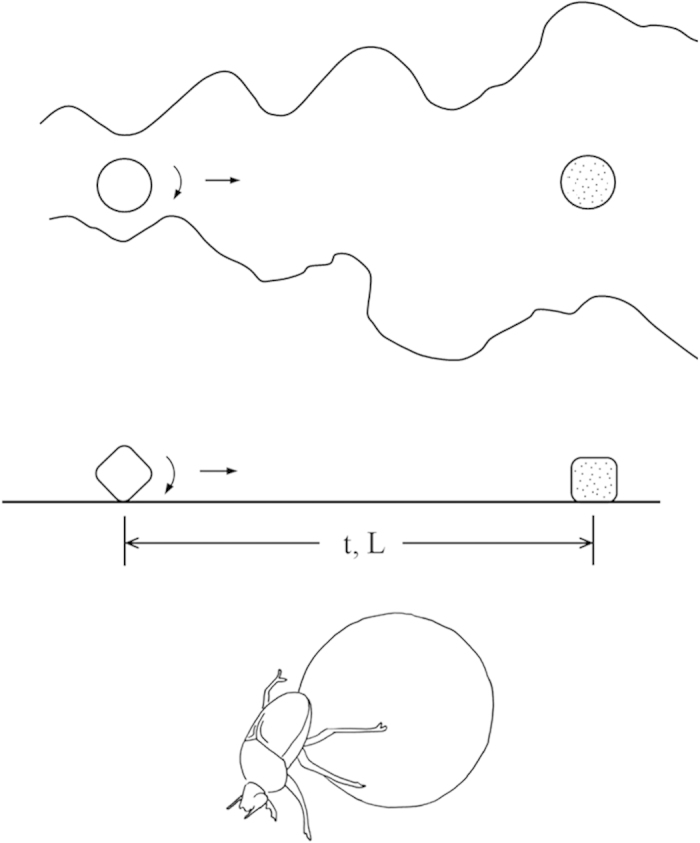
Life span (t) and travel (L) in rolling stones and eddies of turbulence.

Think of a stone rolling on a horizontal plane (Fig. 1, middle). Its mass is M, and the horizontal friction force that slows it down is F ~ μ_f_Mg, where μ_f_ is the friction coefficient. There is friction between the rolling stone and the plane, because the stone has rough features. No stone is a perfect sphere, although all rolling stones evolve toward becoming spherical over time.

The initial velocity of the stone is V. Because of friction, the velocity decreases to zero at the end of the rolling distance L, after the rolling time t. These two measures, t and L, are the life span and life travel of the stone. How large are they?

The life of the stone is a consequence of the initial kinetic energy of the rolling stone, which is 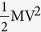. Here we neglect numerical factors of order 1, and recognize MV^2^ as the order of magnitude of the initial kinetic energy of the system. The kinetic energy is transformed completely into work (F · L), which is dissipated through friction and rejected as heat to the ambient. From the balance between M V^2^ and F L, we discover that L scales as V^2^/(μ_f_g), and t scales as V/(μ_f_g).

Why is a bigger stone rolling faster and farther? The reason is that a rolling stone is far from spherical. It hurtles up and down as it rolls, while its center of mass describes a wiggly trajectory with an amplitude proportional to the deviations of its shape from the ideal spherical shape. The length scale of such deviations is the same as the body length scale, namely D ~ (M/ρ_s_)^1/3^, where ρ_s_ is the density of the stone (alternatively, M ~ ρ_s_D^3^). Each roll is a falling-forward motion: the forward speed of this motion is the Galilean speed associated with falling from a height of order D, therefore V ~ (gD)^1/2^, which also means that V is proportional to M^1/6^. This is why bigger stones travel faster.

Next, we return to the formulas for t and L, and use g^1/2^(M/ρ_s_)^1/6^ in place of V. We obtain L ~ (M/ρ_s_)^1/3^/μ_f_ and t ~ (M/ρ_s_)^1/6^/(μ_f_g^1/2^). With these formulas we can predict several features of rolling stones of all sizes:

1. Bigger stones should travel farther and “live” longer. Note the proportionalities between L and M^1/3^, and between t and M^1/6^.
2. The number of rolls (N) is independent of body size. Note the time scale of one roll (one fall forward), t_r_ ~ D/V, therefore N ~ t/t_r_ ~ 1/μ_f_, which is a constant.
3. The life span t is proportional to the square root of the life travel L, and the ratio t/L^1/2^ is independent of body size. This is quite similar to what was shown for animals (t ~ M^1/4^, L ~ M^5/12^) and vehicles^19^.

Hidden in these relations is the evolutionary tendency of the design of the rolling stone. The observed is obvious: any stone that rolls becomes more round. We see this during the process of rolling steel balls between two abrasive surfaces until they become perfect spheres to be used in ball bearings. The evolution is toward looking more like a sphere, and this means that the coefficient of friction μ evolves naturally toward lower values over time.

The rolling stone that evolves toward smaller μ_f_ values is a design that evolves toward greater life span (t) and greater life travel (L), because of the proportionalities t ~ 1/μ_f_ and L ~ 1/μ_f_. This tendency toward easier and greater access of movement, via design evolution, unites all the moving bodies discussed in the present report and ref. 19, animate and inanimate. This is why human life evolved from movement without wheels to movement with wheels, not vice versa^22^.

The dung rolled by the beetle comes close to a perfect sphere (Fig. 1, bottom). The dung and the beetle constitute a rolling stone with a “motor” inside, which is analogous to the animal (or vehicle) viewed as a packet of river water with a propulsion system inside.

**Figure 2:**
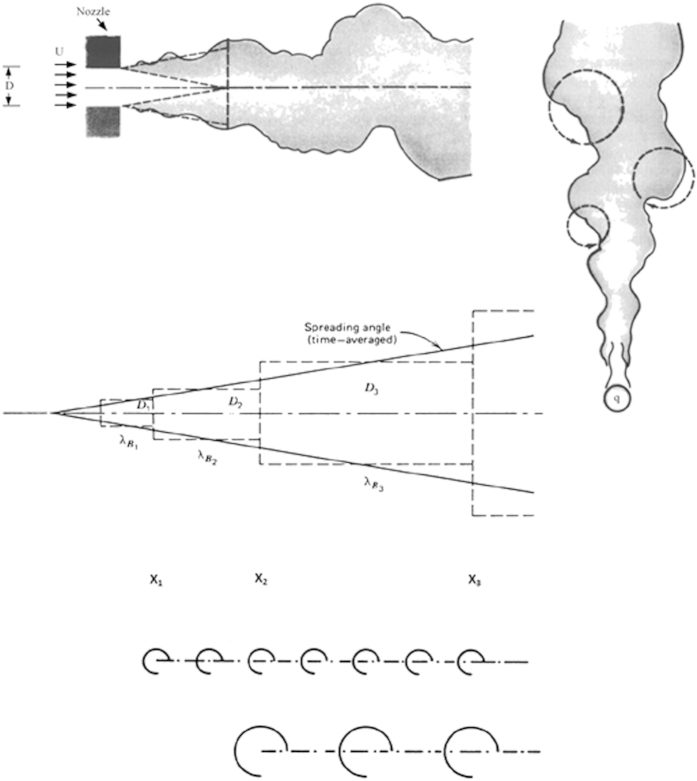
Turbulent jets, plumes and shear layers: the time averaged flow occupies a cone or wedge with constant angle of roughly 20°. The stepwise structure of the buckling flow and eddy generation mechanism are predicted from the constructal law. Smaller eddies are generated more frequently from near the origin of the flow region. Larger eddies are generated less frequently from farther downstream. All eddies die downstream because of the viscous dissipation of their kinetic energy.

An eddy rolling in a turbulent flow has the same life span, life travel and death as the rolling stone and all the other mass movers discussed previously. Review the eddy generation phenomenon illustrated in Fig. 2, and imagine that you move with the frame of one of those eddies moving downstream. The eddy is a fluid eye that rotates inside a fluid socket. The rotational kinetic energy of the eddy is dissipated through friction against the socket until the rotation stops. When the rotation stops, the eddy is dead. It does not exist anymore. It is no longer distinct.

In the simplest description, the eddy has two scales, a diameter D and a rolling peripheral speed V. The latter is dictated by the mixing region in which the eddy is an inhabitant—wedge, cone, jet or plume. Here we treat V as a known external parameter, which is independent of the eddy size. Said another way, the turbulent flow along the mixing region is populated with eddies of many sizes, and in the finite-length section of the flow (in which we ride on the frame of the eddy) the order of magnitude of V does not change. The rotational kinetic energy of the eddy is of the order of MV^2^, where the eddy mass M is of the order of ρD^3^, and ρ is the fluid density.

The kinetic energy is dissipated entirely through rolling friction, which in fluid flow is a shearing motion between small sleeves of fluid rotating inside larger sleeves. The peripheral (tangential) friction force is F ~ τD^2^, where D^2^ is the order of magnitude of the eddy surface, and the viscous shear stress is of the order of τ ~ μV/D, where μ is the fluid viscosity. The dissipation rate is F · V: this is the rate at which the kinetic energy decreases because of friction. Dividing the kinetic energy by the dissipation rate, we discover the life span of the eddy as a rolling carrier of mass: t ~ M/(μD), which is the same as t ~ D^2^/ν, where ν is the fluid kinematic viscosity, μ/ρ.

The first conclusion is that bigger eddies should persist longer in time. The eddy life span is proportional to D^2^, which means M^2/3^. The lifetime travel of the eddy is to the downstream distance L ~ Vt ~ VD^2^/ν. This leads to the second conclusion: bigger eddies should travel farther. The formulas discovered for eddy life span (t) and life travel (L) do not change at all if we replace the eddy eye model (one dimension, D) with a rolling pin model, that is a rolling cylinder with diameter D and an axial length greater than D.

The dying eddy disappears as a macroscopically discernible body, but the shapeless movement (by diffusion) lingers for some time in its place because of the movement of the body that once was. To use Eames’s metaphor^23^ about bubbles and droplets that vanish due to condensation and evaporation, “death” is disappearing bodies and ghost vortices that eventually vanish.

How many times does the eddy roll before it dies? The time scale of one roll is t_r_ ~ D/V. The number of rolls en route to death is N ~ t/t_r_ ~ VD/ν. The surprise is that this number is not only a constant, independent of eddy size (it is the “local” Reynolds number VD/ν, which marks the transition, the eddy birth^24^), but it is a constant that has the same order of magnitude for all living and dying eddies. That number—the number of rolls over a lifetime—is of the order of 10^2^ for all eddies (cf. Table 6.2 in ref. 24).

## Discussion

The life span phenomenon is everywhere, not just in animals, vehicles, rivers, jets and plumes^19^. Every rolling stone and turbulent eddy has it. A rolling stone exhibits the same life features of all the mass movers united by this theory. Bigger stones roll farther, their movement lasts longer, and their number of rolls (their “breaths”, or “heartbeats”) is constant, independent of size. This trend is in accord with observations, because we are all familiar with images of big stones that roll fast. This is why boulders rolling down the road following a rock slide cannot be outrun by man. This is also why the running soccer player catches up with the rolling ball.

These three characteristics, life span, life travel and the constancy of the number of heartbeats and breaths of bodies that move mass, unites the animal, the eddy, and the rolling stone. Life is the changing movement, in both time and in space: from birth to death, and from birthplace to the end of travel, which then becomes the birthplace of a new moving thing. The dung beetle, with its successors growing out of the dung ball, illustrates the continuity of the driven movement (Fig. 1, bottom). This concept is present in many religions, West and East, starting with ancient Egypt. What moves—the complicated immensity of the biosphere, atmosphere, hydrosphere and lithosphere—moves with organization and evolution.

## Additional Information

**How to cite this article**: Bejan, A. Rolling stones and turbulent eddies: why the bigger live longer and travel farther. *Sci. Rep.* **6**, 21445; doi: 10.1038/srep21445 (2016).
